# A case report of successful vaginal delivery in a patient with severe uterine prolapse and a review of the healing process of a cervical incision

**DOI:** 10.1016/j.crwh.2021.e00375

**Published:** 2021-12-14

**Authors:** Jota Maki, Tomohiro Mitoma, Sakurako Mishima, Akiko Ohira, Kazumasa Tani, Eriko Eto, Kei Hayata, Hisashi Masuyama

**Affiliations:** Department of Obstetrics and Gynecology, Okayama University Graduate School of Medicine, Dentistry and Pharmaceutical Sciences, 2-5-1 Shikata-cho, Kita-ku, Okayama 700-8558, Japan

**Keywords:** Suture techniques, Scars, Complete uterine prolapse, Pregnancy, Vaginal delivery

## Abstract

**Background:**

The incidence of severe uterine prolapse during childbirth is approximately 0.01%. Moreover, to the best of our knowledge, no reports detail the healing process of the cervix during uterine involution. This report describes successful vaginal delivery and the healing process of postpartum uterine prolapse and cervical tears in a patient with severe uterine prolapse.

**Case presentation:**

A patient in her 40s (gravida 3, para 1, abortus 1) with severe uterine prolapse successfully delivered a live female baby weighing 3190 g at 38 + 5 weeks of gestation by assisted vaginal delivery. Uterine prolapse had improved to approximately 2° by 2 months postoperatively. On postpartum day 4, during the healing process of cervical laceration, the thread loosened in a single layer of continuous sutures due to uterine involution, and poor wound healing was observed. The wound was subsequently re-sutured with a two-layer single ligation suture (Gambee suture + vertical mattress suture). However, on postpartum day 11, a large thread ball was hindering the healing of the muscle layer, which improved with re-suturing.

**Conclusion:**

Although vaginal delivery in a patient with severe uterine prolapse is possible in some cases, the cervix should be sutured, while considering cervical involution after delivery.

## Highlights

• Pregnancies with complete uterine prolapse are exceedingly rare.
• When suturing the uterus, involution after delivery should be considered.
• This is the first report on the healing process of cervical canal lacerations.

## 1 Introduction

Pregnancies complicated by uterine prolapse are rare, and cases of severe uterine prolapse are rarer [1]. Recommendations regarding the management of this infrequent but potentially fatal condition are scarce [1]. Pregnancies with severe uterine and cervical prolapse have been reported; however, the delivery method is complicated in most cases, and few reports of successful vaginal delivery despite severe cervical edema have been reported in the literature [2]. This report describes a case of vaginal delivery in a patient with severe uterine prolapse and presents the first description of the healing process after a Duhrssen incision performed on a prolapsed cervix.

## 2 Case Presentation

A 43-year-old woman, gravida 3, para 1, abortus 1, implanted with frozen-thawed embryos was diagnosed with high-risk pregnancy at her previous hospital. Her obstetric history included a term, vaginal singleton delivery (3298 g, male neonate) 4 years earlier, which was complicated by a retained placenta. The placenta was removed manually. Thereafter, no uterine prolapse was observed.

**Fig. 1:**
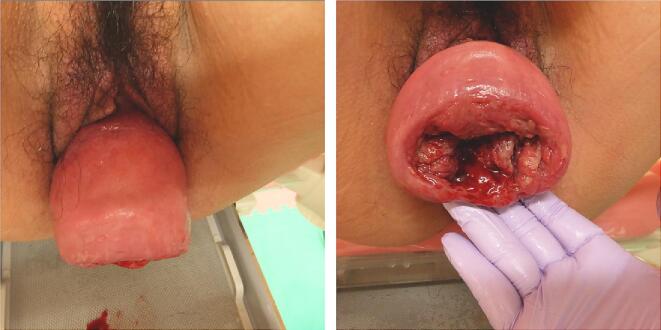
Photographs at the onset of uterine prolapse at 36 weeks of gestation.

**Fig. 2:**
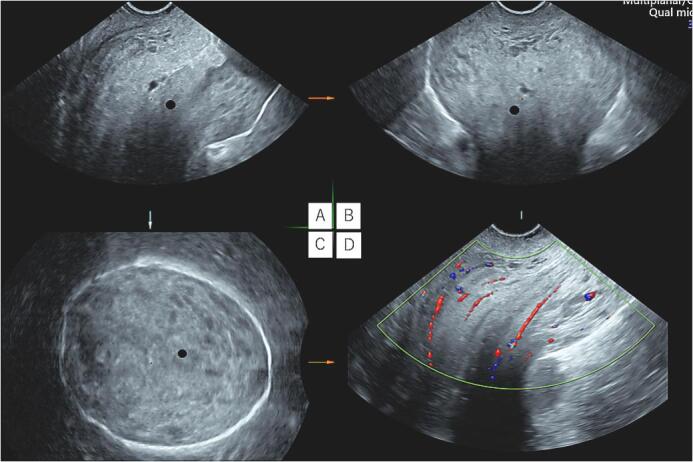
Cervical edema and cervical length of 8.8 cm, color Doppler with 3D transvaginal ultrasonography. A, Sagittal section; B, Coronal section; C, Transverse section; D, Color Doppler.

She had been undergoing treatment for hypertension and dermatomyositis since 2018. She had been prescribed nifedipine CR 20 mg/day for the hypertension and steroids (prednisolone) 4.0 mg/day and tacrolimus 3.4 mg/day for the dermatomyositis. As the symptoms of dermatomyositis improved in 2019, the pregnancy was approved. She had also undergone sub-plasmalemmal myomectomy 10 years previously. She was otherwise fit and had a normal body mass index; she did not smoke and had no allergies. During the index pregnancy, at the 19-week check-up, her cervix was found to have descended. Subsequent regular ultrasound scans at 24, 26, 30, 32, and 34 weeks of gestation demonstrated normal fetal morphology, with normal and concordant fetal growth. An ultrasound scan at 36 weeks showed that the cervical canal was very long (8.8 cm) and closed (Fig. 1). A 95-mm self-removable donut-shaped pessary (donut support for 3° prolapse/procidentia; CooperSurgical Milex™, 75 Corporate Drive Trumbull, CT 06611 USA) appeared to drop out spontaneously. Color Doppler imaging with 3D transvaginal ultrasonography revealed cervical edema (Fig. 2). Color Doppler examination did not reveal any obvious blood flow. She was admitted to hospital at 37 + 2 weeks due to a worsening skin rash over the perineum and difficulty in washing. Her blood pressure was stable, her general condition was good, and there were no abnormal clinical or biochemical findings suggestive of an infection.

In the pre-induction state of the cervix, the Bishop scoring system showed that the cervical os was dilated by 4 cm, effaced 0%, station −3, soft, and anterior 7 points. Induction with oxytocin was commenced at 38 weeks and 4 days of gestation for planned delivery, but there were no effective contractions. The next day, the cervix was dilated by 6 cm and effaced 30%.

**Fig. 3:**
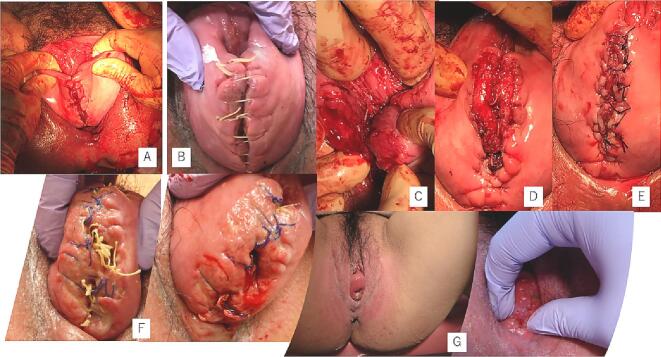
Healing process of cervical laceration and cervical canal prolapse. A: Cervical laceration suture surface on the day of delivery. B: On postpartum day 4, suture failure was noted due to a continuous 1-layer suture (0 mesh thread (Vicryl®), absorbable thread). C: On postpartum day 4, stitches were removed and debridement was performed. D: On postpartum day 4, re-suture and first-layer Gambee suture (2–0 mesh thread, absorbable (Vicryl®)) were performed. E: On postpartum day 4, re-suture and second-layer horizontal mattress suture (2–0 mesh thread, absorbable (Vicryl®)) were performed. F: On postpartum day 11, the thread had formed into a ball, which interfered with viability. The wound was re-sutured using a single ligation due to suture failure (3–0 mesh thread, absorbable (Vicryl®)). G: On postpartum day 28, uterine prolapse improved to 2°–3°, and the wound became viable.

With the patient's consent, we performed an artificial rupture of the membranes, which led to labor progression. After an intramuscular injection of butyl scopolamine, her cervix was softened for a short time. The cervix was completely prolapsed outside the vagina, but the station had reached +1 in relation to the pubic symphysis. The 95-mm donut-type pessary was easily removed from the soft birth canal, and vacuum extraction delivery (Kiwi®) was deemed feasible. Cervix-holding forceps were used, and a Duhrssen incision was made under visualization as labor progressed. A cervical Duhrssen incision of approximately 5 cm was made, and the length of the incision was extended to 10 cm. We did not perform a perineal incision. After three traction cycles, a live female neonate weighing 3190 g was delivered, with Apgar scores of 8/9 (1st and 5th minutes) and a pH of 7.319. The delivery was complicated with a retained placenta and massive postpartum hemorrhage (PPH), with blood loss of 2500 mL. The cervix was sutured, as usual, using 0 Vicryl® sutures in one continuous layer from 5 mm above the upper edge of incision, and the surface of the suture appeared to be fine. However, on discharge examination on the 4th day post-delivery, a complete breakdown of the repair of approximately 5 cm of the cervical canal was identified at the introitus. After she was returned to the operating theatre, debridement was performed; the first layer was sutured with a single ligation using the Gambee suture (2–0 Vicryl®), and the second layer was sutured with an end-to-end single ligation using the mattress suture technique (2–0 Vicryl®). On the 7th day after re-suturing (11th day after delivery), the uterus was markedly involuted, and uterine prolapse somewhat improved, but the ligature threads were collected in one place and became a single ball of threads. All sutures were removed, and the wound was re-sutured with two Z-sutures using a 3–0 Vicryl® thread. Sutures were removed 1 month postpartum, and complete wound healing was confirmed. Two months post-surgery, uterine prolapse had improved to a 2° prolapse. The healing process of cervical laceration and cervical canal prolapse is shown in Fig. 3.

## 3 Discussion

Uterine and cervical prolapse are rare in pregnancy. Uterine prolapse during pregnancy is a precarious event, with an incidence of 1 in 10,000–15,000 pregnancies [3]. It can be associated with minor cervical desiccation and ulceration or devastating maternal fatalities. Complications include urinary retention, preterm labor, premature delivery, fetal demise, maternal sepsis, and urinary retention [1].

Prolapse development in pregnancy is likely due to an escalation of the physiological changes in pregnancy, leading to a weakening of pelvic organ support [4]. Increased cortisol and progesterone levels during pregnancy may contribute to uterine relaxation. In our case, the patient did not have any congenital risk factors. However, she had developed dermatomyositis after her last delivery and had been prescribed prednisolone 4 mg/day which she had taken consistently since before pregnancy. In a previously reported case study, pessary insertion resulted in the temporary improvement of uterine prolapse [5,6], but, in our case, a 95-mm donut pessary (CooperSurgical Milex™) easily slipped out of the soft birth canal. According to a case review of pregnancies with uterine prolapse reported by Kana et al. [7], since 1997, vaginal delivery could be performed in only 2 of 16 patients with cervical edema, and none of them was diagnosed with complete uterine prolapse. Jeong et al. [8] reported that, since 2002, only 1 of 14 patients with uterine prolapse was able to be delivered vaginally. These results suggest that this study reports a valuable case of vaginal delivery. In fact, the two cases reported recently were both delivered by cesarean section [9,10].

We performed vaginal delivery, and a Duhrssen incision was stitched with minimal blood loss. In active labor, a prolapsed cervix that is enlarged and edematous can be managed with a topical concentrated magnesium solution to treat cervical dystocia and lacerations [11]. Verma et al. reported that two incisions approximately 3 cm long each were made at 2 o'clock and 10 o'clock positions on the cervix using tissue-cutting scissors [12]. In contrast, our approach involves using one incision that is approximately 5 cm long at the 6 o'clock position on the cervix using tissue-cutting scissors. Cervical laceration suturing was performed, and we observed that a single layer of continuous sutures had lost tension and was loose due to the significant involution of the cervix. Moreover, the external os was found outside the vagina; thus, the wound could not be covered, and thread traction could not prevent incision separation at 6 o'clock. After debridement, Gambee sutures and horizontal mattress sutures were used to re-suture the wound, but on postpartum examination on day 11, the ligature thread from the single ligation suture had formed a ball of threads. We re-sutured the wound using 3–0 Vicryl® sutures, and the wound was stabilized. The cervix was edematous and markedly dilated; therefore, any sutures are likely to be extruded as the cervix involutes and the edema improves. A technique that results in the end-to-end apposition of the incision is more likely to succeed as it allows for clot formation and healing by primary intention with less reliance on suture tension. Nevertheless, the majority of such cases have been managed by cesarean section. This vaginal delivery resulted in a prolonged process of labor induction, cervical incision (which likely induced long-term cervical incompetence), three trips to the operating room, a retained placenta, and a massive PPH; however, direct causality of the last two factors is unknown. Avoiding cesarean section in this case resulted in an unavoidable burden on the patient after delivery. Although we do not necessarily recommend vaginal delivery, we report it as an option. An extensive literature search listing the keywords “healing process, cervical canal lacerations, single ligation suture, and continuous sutures” revealed that this is the first report on the healing process of cervical canal lacerations.

## 4 Conclusion

Vaginal delivery of a pregnancy complicated by severe uterine prolapse is possible in some cases. Given the paucity of data on the management and pregnancy outcomes of patients with uterine anomalies, such cases should be considered high risk, and individualized care should be given to patients to optimize outcomes. Moreover, when suturing the uterus cervix, uterus cervical involution after delivery should be considered. This case report adds to the literature as it highlights the management of a rare obstetric condition.

The following is the supplementary data related to this article.

Video 1 Video of assisted vaginal delivery and salvage from cervical dystocia in uterocervical prolapse: Duhrssen incision Video 1

Supplementary material Image 1

## Contributors

Jota Maki was responsible for the conceptualization of the case report, was the main author, conducted the literature review, and drafted the manuscript, and was involved in the patient’s treatment, from outpatient care to hospitalization and discharge.

Tomohiro Mitoma was responsible for the conceptualization of the case report, was involved in the case, and reviewed and revised the manuscript.

Sakurako Mishima was responsible for the conceptualization of the case report, and reviewed the draft.

Akiko Ohira was responsible for the conceptualization of the case report, and reviewed the draft.

Kazumasa Tani was responsible for the conceptualization of the case report, and reviewed the draft.

Eriko Eto was responsible for the conceptualization of the case report, was involved in the case, and reviewed and revised the manuscript.

Kei Hayata was responsible for the conceptualization of the case report, and reviewed the draft.

Hisashi Masuyama was responsible for the conceptualization of the case report, was involved in the case, and reviewed and revised the manuscript.

All authors approved the final version of the paper and take full responsibility for the work.

## Funding

This work did not receive any specific grant from funding agencies in the public, commercial, or not-for-profit sectors.

## Patient consent

The patient provided informed consent for the publication of this report and all accompanying images.

## Provenance and peer review

This case report was not commissioned and was peer reviewed.
