# Supplementary material for: A case report of successful vaginal delivery in a patient with severe uterine prolapse and a review of the healing process of a cervical incision
Source: Case Rep Womens Health. 2021 Dec 14;33:e00375. doi: 10.1016/j.crwh.2021.e00375 (PMC8703083; doi:10.1016/j.crwh.2021.e00375)
Supplement: Supplementary file 2 — Supplementary material [file mmc2.pdf]

CARE case report guidelines

CARE Checklist of information to include when writing a case report

(cc) BY-NC-ND

Topic	Item	Checklist item description	Reported on Line
**Title**	1	The diagnosis or intervention of primary focus followed by the words "case report"	1
**Key Words**	2	2 to 5 key words that identify diagnoses or interventions in this case report, including "case report"	31
**Abstract (no references)**	3a	Introduction: What is unique about this case and what does it add to the scientific literature?	55-57
	3b	Main symptoms and/or important clinical findings	74-78
	3c	The main diagnoses, therapeutic interventions, and outcomes	84-113
	3d	Conclusion—What is the main "take-away" lesson(s) from this case?	159-164
**Introduction**	4	One or two paragraphs summarizing why this case is unique (**may include references**)	117,154
**Patient Information**	5a	De-identified patient specific information.	60
	5b	Primary concerns and symptoms of the patient.	61
	5c	Medical, family, and psycho-social history including relevant genetic information	62-69
	5d	Relevant past interventions with outcomes	63-64
**Clinical Findings**	6	Describe significant physical examination (PE) and important clinical findings.	71-73
**Timeline**	7	Historical and current information from this episode of care organized as a timeline	72-113
**Diagnostic Assessment**	8a	Diagnostic testing (such as PE, laboratory testing, imaging, surveys).	75-78
	8b	Diagnostic challenges (such as access to testing, financial, or cultural)	78-81
	8c	Diagnosis (including other diagnoses considered)	74
	8d	Prognosis (such as staging in oncology) where applicable	55,112
**Therapeutic Intervention**	9a	Types of therapeutic intervention (such as pharmacologic, surgical, preventive, self-care)	82-110
	9b	Administration of therapeutic intervention (such as dosage, strength, duration)	82-110
	9c	Changes in therapeutic intervention (with rationale)	102-110
**Follow-up and Outcomes**	10a	Clinician and patient-assessed outcomes (if available)	111-113
	10b	Important follow-up diagnostic and other test results	111-113
	10c	Intervention adherence and tolerability (How was this assessed?)	110-111
	10d	Adverse and unanticipated events	102-103
**Discussion**	11a	A scientific discussion of the strengths AND limitations associated with this case report	136,146,151
	11b	Discussion of the relevant medical literature **with references**.	131-136
	11c	The scientific rationale for any conclusions (including assessment of possible causes)	122,135
	11d	The primary "take-away" lessons of this case report (without references) in a one paragraph conclusion	160-162
**Patient Perspective**	12	The patient should share their perspective in one to two paragraphs on the treatment(s) they received	55-57
**Informed Consent**	13	Did the patient give informed consent? Please provide if requested	Yes ☑ No ☐
